# Locomotion and kinematics of arachnids

**DOI:** 10.1007/s00359-021-01478-2

**Published:** 2021-03-18

**Authors:** Jonas O. Wolff

**Affiliations:** 1 Zoological Institute and Museum, University of Greifswald, Loitzer Str. 26, 17489 Greifswald, Germany; 2 Department of Biological Sciences, Macquarie University, Sydney, NSW 2109 Australia

**Keywords:** Octopedal locomotion, Animal biomechanics, Animal performance, Movement ecology, Kinematic analysis

## Abstract

A basic feature of animals is the capability to move and disperse. Arachnids are one of the oldest lineages of terrestrial animals and characterized by an octopodal locomotor apparatus with hydraulic limb extension. Their locomotion repertoire includes running, climbing, jumping, but also swimming, diving, abseiling, rolling, gliding and -passively- even flying. Studying the unique locomotor functions and movement ecology of arachnids is important for an integrative understanding of the ecology and evolution of this diverse and ubiquitous animal group. Beyond biology, arachnid locomotion is inspiring robotic engineers. The aim of this special issue is to display the state of the interdisciplinary research on arachnid locomotion, linking physiology and biomechanics with ecology, ethology and evolutionary biology. It comprises five reviews and ten original research reports covering diverse topics, ranging from the neurophysiology of arachnid movement, the allometry and sexual dimorphism of running kinematics, the effect of autotomy or heavy body parts on locomotor efficiency, and the evolution of silk-spinning choreography, to the biophysics of ballooning and ballistic webs. This closes a significant gap in the literature on animal biomechanics.

## Background

Legged locomotion is a common feature of most terrestrial animals. One of the oldest lineages of terrestrial animals are the arachnids. Extant arachnids conquered nearly all types of terrestrial ecosystems and play significant ecological roles as predators and parasites. They are capable of a diversity of locomotory modes, including running, climbing, jumping, but also swimming, diving, rolling and -passively- even flying. Arachnid locomotor performance is often impressive. The fastest arachnids are cursorial hunters, such as wandering spiders, solifuges and predatory mites—the latter of which may reach speeds of up to 100 body lengths per second by super-fast muscle contractions (Wu et al. 2010; Spagna and Peattie 2012). Discoveries of outstanding locomotory features in arachnids are ongoing: Recent research found an extreme turning speed of some spiders with laterigrade leg configuration (Zeng and Crews 2018), gliding locomotion in canopy spiders (Yanoviak et al. 2015), somersaulting escape movements in desert huntsmen (Jäger 2014), sailing behaviour on water bodies (Hayashi et al. 2015), and ultra-fast power-amplified movements in the mouth parts of trap-jaw spiders (Wood et al. 2016) and some harvestmen (Wolff et al. 2016). In spiders, the production of silk further extends movement capacities. For instance, the use of a ‘kite’ from charged, floating silk lines is a frequent and efficient mechanism for long-distance dispersal. Such ballooning spiders have been found as high as one thousand metres above the ground (Glick 1939). Abseiling and bridging behaviour facilitates movement through the three-dimensional space and opens up new niches (Gregorič et al. 2011). Some spiders also use silk for elastic energy storage to build special spring-loaded traps that pull the prey into the vicinity of the resting spider (Argintean et al. 2006; Greco and Pugno 2021).

Research on arachnid locomotion started with detailed comparative anatomical investigations of their locomotory apparatus (reviewed in Shultz 1989). These studies highlighted that extensor muscles are absent in most arachnid joints, leading to speculations about the biomechanical function of arachnid legs (Petrunkevitch 1909; Ellis 1944). This triggered the integrative study of arachnid locomotion as a focal topic. For instance, the combination of morphological, physiological and biomechanical methods helped to test hypotheses on the hydraulic function and the role of hemolymph pressure in arachnid leg extension (Parry and Brown 1959; Shultz 1991; Sensening and Shultz 2003). Others focused on the sensory control of locomotion (Blickhan and Barth 1985), or on respiratory physiology and the question why spiders show such rapid fatigue (Prestwich 1988; Schmitz 2005). Early on, also the question of how some spiders can scale smooth vertical surfaces with adhesive foot pads have been approached experimentally (Homann 1957), which, however, could not be resolved before the development of modern nano-mechanical measurement techniques (reviewed in Wolff and Gorb 2016).

The advent of high-speed videography brought an important tool for the kinematic study of fast arachnid movements, such as the jumping of salticid spiders (Nabawy et al. 2018) and box mites (Wauthy et al. 1998), or the predatory strikes of spiders (Eggs et al. 2015; Wood et al. 2016; Zeng and Crews 2018) and amblypygids (Seiter et al. 2019). Detailed kinematic studies of running spiders also highly advanced the understanding of the biomechanical function of the arachnid locomotor system and overturned some long upheld beliefs—for instance they questioned hydraulics as the main drive of locomotion in large spiders (Weihmann et al. 2012). The combination of kinematics and fluid mechanics uncovered the mechanisms by which fishing spiders can move on water (Gorb and Barth 1994; Suter et al. 1997) and how spitting spiders eject a sticky web on their prey by passive oscillatory movements of their chelicerae (Suter and Stratton 2009).

More recently, the integration of robotics and material science into the study of arthropod biomechanics brought another leap in the understanding of the arachnid locomotor system and attracted engineers to it. For instance, the underactuated locomotory system of arachnids has been repeatedly picked up by biomimetic robotics (reviewed in Landkammer et al. 2016). Further, the octopedal locomotory mode has been studied as a way to achieve a high stability and robustness of walking in robots when moving through complex terrains (Klaassen et al. 2002; Spagna et al. 2007).

Beyond the biomechanical aspects, arachnid locomotion has also been in the focus of many ecological and behavioural studies, facilitated by the development of computational video tracking methods. This encompasses questions, such as how arachnids navigate through complex environments (reviewed in Gaffin and Curry 2020), disperse (Powers and Aviles 2003), utilize distinct movement patterns for mimicry (Shamble et al. 2017), construct webs (Zschokke and Vollrath 1995), or on the role of locomotor performance for sexual selection (Moya‐Laraño et al. 2009; Prenter et al. 2010). Finally, by integrating the detailed observation of locomotion in extant arachnids with detailed morphological studies, the movements of ancient chelicerates can be reconstructed with modern computational simulation tools, advancing our understanding on the evolution and origins of the peculiarities of arachnid locomotion (Garwood and Dunlop 2014).

These examples showcase the richness of the research on arachnid locomotion and its far reach into various disciplines. At the same time, it shows that an understanding of locomotor function is most successful by the application of elaborate interdisciplinary approaches, which makes this topic both appealing, innovative and challenging.

The aim of this special issue is to display the state of research on arachnid locomotion, linking physiology and biomechanics with ecology, ethology and evolutionary biology. The idea arose in the context of the 20th International Congress of Arachnology in Golden, Colorado in 2016. Linda Rayor, one of the organizers and also a contributor to this special issue, asked me to compile a symposium on arachnid locomotion, which resulted in a vivid full-day session. Before organizing this symposium, I was not aware of how active this field has become in recent times. Clearly, what was missing was a synthesis of this advancing field that due to its cross-disciplinary nature and diverse foci was communicated highly scattered across very different journals and communities. I thank Friedrich Barth for inviting me to edit this special issue for the Journal of Comparative Biology A. Especially I thank all authors and reviewers who contributed their research and ideas to this special issue, making it a significant and representative reference of the field.

The content is structured in three topic blocks, each containing five articles. Note that these blocks do not represent stringent categories and the content is often relevant for diverse fields.

## Kinematics of arachnid movements

Bøhm et al. (2021) explored the scaling of locomotor speed and limb kinematics in wandering spiders with a size range spanning three orders of magnitude. Using a cutting-edge automated markerless tracking approach, they measured limb flexion and extension speeds and compared them with spider mass. Their data does not support the hypothesis that hydraulic leg extension poses a limit to maximal running speed. Telheiro et al. (2021) studied the running kinematics in fat-tailed scorpions. These arachnids bear a massive telson that is elevated in a defensive posture. The study revealed that the defensive posture changes the location of the centre of mass and that the scorpions deploy a modified gait to stabilize their run. Weissbach et al. (2021) describe the mobility of the spider’s spinnerets and their fine-tuned movements during the production of cribellar capture threads—adhesive mats consisting of hundreds of nano-fibres. In Wolff (2021), I report on a comparative study of spinneret choreographies during the production of thread anchorages. I showed that there was a repeated trend towards high spinning speeds in spider evolution and I reconstructed the evolutionary change of kinematic patterns by applying a geometric morphometric approach to spinning track shapes. Brandt et al. (2021) described the jump kinematics of small salticid spiders. They deployed a deep learning-based markerless tracking approach to observe the use of different limbs during take-off and found that jumps are mainly powered by the third legs in their study species. Their study also inspired the cover image of this special issue.

## Biomechanics of arachnid movements

Goetzke and Federle (2021) report on the role of adhesive foot pads to avoid slipping during jumping spider take-off. Using a combination of kinematic, morphological and experimental approaches, they showed that different parts of the footpads are used either for pushing or for pulling and their fine-tuned use enables both a secure foothold and a quick release of the grip during pre-jump acceleration. Silva et al. (2021) review the state of knowledge on the biomechanics and functional morphology of hairy adhesive footpads and the kinematics of walking and climbing in the large theraphosid tarantulas (bird-eating spiders). Blickhan et al. (2021) review the methodology to measure strains in the exoskeleton of arachnids and how it helped to understand the sensory control of spider locomotion and the avoidance of cuticle overload during powerful movements. Challita et al. (2021) studied the dynamics of the special webs built by so-called slingshot spiders. The ballistic movements of these webs are impressive in their extreme acceleration and sudden, damped stop, and the team developed a mathematical model to explore the underlying biomechanics. They found that the special conical web architecture permits it to act both as an elastic spring and a shock absorber, enhancing prey capture successes while avoiding web breakage and spider injury. Cho (2021) reviews the knowledge about the biophysics of ballooning behaviour in spiders. The air-based dispersal of spiders already fascinated Darwin and it is still poorly understood how spiders of up to 150 mg can get airborne using nano-scale silk lines. Cho critically discusses the roles of thermal convection, wind turbulence and electromagnetic forces as the driving forces of spider ballooning.

## Neurophysiology and behavioural ecology of arachnid movements

Barth (2021) summarizes the state of knowledge on the neural control of spider movements in locomotion, prey capture and reproduction. Escalante et al. (2021) studied the effect of autotomy on respiratory rates in escaping harvestmen. They let the intact and de-legged harvestmen run on a treadmill inside a flow-through respirometer and found that leg loss increased the oxygen consumption and led to faster exhaustion, limiting the efficiency of autotomy as a means of predation avoidance. Hurst and Rayor (2021) comparatively studied the locomotor performance of female and male huntsman spiders to understand the role of sexual dimorphism seen in the body-to-leg ratios of most spiders. They found while the absolute speed did not differ between sexes, male locomotion was more energy efficient. Buzatto et al. (2021) review the knowledge on mygalomorph spider dispersal and relate it to the distribution ranges modelled from faunistic data. Most of these spiders are ground-dwelling and poor dispersers, but some lineages have evolved ballooning behaviour and the authors showed that this significantly extended the geographical species ranges. Rosales-Garcia et al. (2021) tracked the movements of small kleptoparasitic spiders in giant orb webs. They showed that the kleptoparasites move more carefully in the inner part of the web, where the host spider resides, but also visit this area more often than locations in the web periphery.

This topical issue is a compendium of current knowledge on arachnid locomotion. I hope the reader will find it a useful resource, a long-lasting reference on the topic and an inspiration to contribute to the vivid research on this exciting topic.
